# The Heterogeneity of Ornamental Plants in Nurseries Increases the Chance of Finding New Hosts for *Phytophthora*

**DOI:** 10.3390/jof11030187

**Published:** 2025-02-27

**Authors:** Alejandro Soto-Plancarte, Marlene Díaz-Celaya, Gerardo Rodríguez-Alvarado, Yolanda Leticia Fernández-Pavía, Hilda Victoria Silva-Rojas, Martha Elena Pedraza-Santos, Rafael Salgado-Garciglia, Tyler Baldwin Bourret, Sylvia Patricia Fernández-Pavía

**Affiliations:** 1 Laboratorio de Patología Vegetal, Instituto de Investigaciones Agropecuarias y Forestales, Universidad Michoacana de San Nicolás de Hidalgo (UMSNH), km 9.5 Carr. Morelia-Zinapécuaro, Tarímbaro 58880, Michoacán, Mexico; alexsotoppv@gmail.com (A.S.-P.); marlenediazcelaya@gmail.com (M.D.-C.); gra.labpv@gmail.com (G.R.-A.); 2 Programa de Edafología-Nutrición Vegetal, Colegio de Postgraduados, Campus Montecillo, km 36.5 Carr. México-Texcoco, Montecillo, Texcoco 56264, Estado de México, Mexico; yletif@yahoo.com.mx; 3 Producción de Semillas, Colegio de Postgraduados, Campus Montecillo, km 36.5 Carr. México-Texcoco, Montecillo, Texcoco 56264, Estado de México, Mexico; hsilva@colpos.mx; 4 Facultad de Agrobiología, Universidad Michoacana de San Nicolás de Hidalgo, Paseo Lázaro Cárdenas esq. Berlín, Colonia Viveros, Uruapan 60090, Michoacán, Mexico; marelpesa@yahoo.com.mx; 5 Instituto de Investigaciones Químico-Biológicas, Universidad Michoacana de San Nicolás de Hidalgo, Edif. B-3, Ciudad Universitaria, Morelia 58060, Michoacán, Mexico; rafael.salgado@umich.mx; 6 Mycology and Nematology Genetic Diversity and Biology Laboratory, United States Department of Agriculture-Agricultural Research Service, 10300 Baltimore Ave, Beltsville, MD 20705, USA; tyler.bourret@usda.gov

**Keywords:** oomycota, *Phytophthora*, potted plants, plant diseases

## Abstract

The production of ornamental plants in Mexico represents a job-generating activity that has grown in recent years; however, it is adversely affected by phytosanitary issues, notably those induced by *Phytophthora*. Studies of *Phytophthora* in ornamental nurseries are scarce in Mexico. The aim in this study was to identify *Phytophthora* species from selected ornamental plant nurseries in Mexico as potential new hosts. Samples of 13 genera diseased plant tissue and soil were collected from eight nurseries in Mexico during 2009–2010. Based on morphology and sequences of ITS rDNA, the 19 isolates obtained were identified as *P. cactorum*, *P. capsici*, *P. cinnamomi*, *P. drechsleri*, *P. kelmanii*, *P. nicotianae*, and *P. tropicalis*. Additional loci were sequenced to support species determinations within the *P. capsici* species complex; some of these isolates could not be confirmed as belonging to any described species, and one appeared to be an interspecific hybrid. This is the first report of *P. kelmanii* in Mexico; this is noteworthy due to being a broad host range, similar to most of the other species encountered. Evidence of nursery-grown plants serving as a *Phytophthora* vector to a home garden has been reported for the first time in Mexico. *Cestrum nocturnum* and *Solanum ovigerum* are new hosts for *Phytophthora* worldwide.

## 1. Introduction

The genus *Phytophthora* comprises more than 250 described species [1,2]. Some of these species cause damage to ornamental nursery plants in various countries worldwide, persisting throughout the year due to the pathogen’s ability to survive in soil, plant residues, containers, and irrigation water [3,4,5,6]. Diseases caused by this genus of oomycetes result in significant losses in the ornamental plant production system. Factors contributing to the presence of these diseases include a diverse range of plants, facilitating cross-infections leading to the emergence of new hosts, as well as poor practices within the nursery. These practices involve substrate and container reuse, maintaining plants in poorly drained surfaces, and disposing of residues from infected plants near production areas [6,7,8]. *Phytophthora* induces symptoms such as stem and root rots, dieback, wilting, chlorosis, and foliar blights [9].

The production and distribution of ornamental plants in Mexico are continuously evolving activities that generate direct employment and, consequently, contribute to social development. In 2022, Mexico produced 5368 million flowers [10], positioning itself among the leading global producers of ornamentals, ranking third worldwide in production area [11]. However, this industry is impacted by diseases caused by pathogens. Most Mexican ornamental plant nurseries present great heterogeneity in regard to hosts. Nevertheless, in Mexico, reports on oomycetes, particularly *Phytophthora*, causing damage to ornamental plants are scarce [12,13,14]. Given Mexico’s megadiverse vegetation [15] and the nationwide distribution of plants produced in different states, *Phytophthora* has the potential of spreading to areas where it was not previously present or affecting new hosts, as discussed in other studies on this pathogen [2,16]. The species with a broad host range are particularly noteworthy, such as *P. cinnamomi*, which is considered the most devastating due to its impact on approximately 5000 hosts [17], or *Phytophthora ramorum*, a species which has moved throughout the world via ornamental nursery production [18,19,20].

The aim of this research was to detect and identify *Phytophthora* isolates from selected ornamental plant nurseries in Mexico as potential new hosts in Mexico and worldwide.

## 2. Materials and Methods

### 2.1. Sample Collection

Sampling was conducted during 2009 and 2010 and included diseased plant tissue and soil from ornamental plants exhibiting symptoms in eight nurseries located in the municipalities of Morelia, Tarímbaro, and Uruapan in Michoacán, as well as from a plant acquired in Mexico City (CDMX) from a nursery in Morelos. In Michoacán nurseries, most plants sold come from different states in Mexico, mainly from Mexico City, State of Mexico, Morelos, and Puebla. Michoacán produces a limited variety of ornamental plant species, including *Cestrum*, *Gardenia*, and *Gazania* [21]. All the sampled plants displayed symptoms of wilting, occasionally exhibiting rot in their stems, crowns, or roots, and dieback.

### 2.2. Obtaining Isolates

#### 2.2.1. Plant Tissue

Sections of tissue measuring 5 to 10 mm^2^ were obtained from the edge of lesions on tissue previously rinsed with tap water. They were disinfested with a diluted solution of commercial chlorine at 10% (a.i. sodium hypochlorite 6%) for 30 s and rinsed twice with sterile distilled water. Tissue sections were placed in Petri dishes with selective NARPH cornmeal medium (natamycin 20 mg L^−1^, ampicillin 27 mg L^−1^, rifampicin 10 mg L^−1^, PCNB 100 mg L^−1^, and hymexazol 75 mg L^−1^). Cultures were incubated at 25 °C in the dark, until a characteristic *Phytophthora* mycelium growth was observed. Pure cultures were obtained from isolates using hyphal tipping [22], grown on cornmeal medium, and stored at 15 °C in microcentrifuge tubes with sterile distilled water.

#### 2.2.2. Soil or Planting Substrate

From the rhizosphere of diseased plants, soil samples weighing 10 g were taken and placed in Petri dishes with 20 mL of sterile distilled water. Disks and complete leaves of azalea (*Rhododendron* sp.) were used as bait tissue and incubated in darkness for 24 to 48 h at 25 °C. Subsequently, bait tissues were washed with sterile distilled water and disinfested with a diluted solution of commercial chlorine at 1% for 30 s, rinsed with sterile distilled water, and dried with sterile absorbent paper. They were then sown on selective NARPH cornmeal medium [23] and incubated at 25 °C in the dark, until characteristic *Phytophthora* mycelium growth was observed. The isolates were stored following the protocol described above.

### 2.3. Morphological Characterization

Isolates were grown on cornmeal, V8-agar (CaCO_3_ 3 g, agar 15 g, Campbell’s^®^ V8 juice 160 mL, distilled water 840 mL), or carrot agar (agar 15 g, diced carrot 50 g, distilled water 1 L). To induce sporulation, mycelium cubes of approximately 5 mm^2^ and 4 to 5 days of growth were cut, covered with sterile distilled water to the edge of the agar, and, for some isolates, soil extract was added when necessary (20 g of soil and 1 L of distilled water, stirred for 3 h and filtered three times with a funnel using a vacuum pump and Whatman filter paper in the following order: 2, GF/A, and GF/C); approximately 5 mL of liquid was added in each plate. The dishes were kept at 25 °C in an incubator (311M, Lab-Line Imperial III, Melrose Park, IL, USA). Sexual structures were induced through crosses for heterothallic species on V8 medium, then incubated at 25 °C in the dark. For homothallic species, direct observations were made. Additionally, green bean–squash medium (339 g green bean–squash Gerber^®^, agar 15 g, and water was added up to 1 L) was used for oospore production with some isolates [23]. Morphological characterization was conducted by observing the presence of asexual structures (sporangia and chlamydospores) and sexual structures (oogonium, antheridium, and oospores) and comparing them with those of described species [1,9,24,25].

### 2.4. Compatibility Type Determination

Reference strains of *P. cinnamomi* A1 (PC3658) and A2 (VAN3) obtained from the collection of the University of California, Riverside, and *P. capsici* A1 and A2 (from chili pepper) from the oomycete collection at the Plant Pathology Laboratory of UMSNH were used. For heterothallic isolates, a mycelial disk from an A1-compatibility-type isolate was placed in a Petri dish containing V8-agar, and a separate dish contained a disk from an A2-compatibility-type isolate. In each dish, a mycelial disk from the isolate whose compatibility type was to be determined was positioned about one centimeter away. The dishes were incubated at 25 °C in the dark for 15 days, and subsequent observation determined oospore formation.

### 2.5. DNA Extraction

A disk from each isolate was placed in Petri dishes (100 × 15 mm) with liquid pea medium (120 g of peas and 1 L of distilled water) and incubated at 25 °C for 1 week. The mycelia were washed with sterile distilled water through a funnel with Miracloth (Sigma-Aldrich, Spruce, St. Louis, MO, USA). Subsequently, the mycelia were wrapped in sterile filter paper and aluminum foil and stored at 4 °C for 24 to 48 h. The mycelia were ground in a sterile mortar with liquid nitrogen and transferred to microcentrifuge tubes and stored at −20 °C. To each tube with ground mycelia, 900 µL of extraction buffer (0.05 M EDTA, 1 M Tris-HCl pH 8.0, 5 M NaCl, 20% SDS, 0.75% β-Mercaptoethanol) preheated to 65 °C was added. Samples were incubated at 65 °C for 1 h. Subsequently, 45 µL of 7.5 M ammonium acetate were added, vigorously mixed for 5 min, and kept on ice for 20 min. Tubes were centrifuged at 13,200 rpm for 15 min. The supernatant was transferred to a tube containing 800 µL of isopropanol; the tubes were manually shaken and kept on ice for 30 min. Samples were centrifuged again at 13,200 rpm for 15 min, and the supernatant was discarded. The pellet was resuspended in 450 µL of TE pH 7.5, and 1 µL of RNase A (20 mg/mL) was added and left overnight at 4 °C. Subsequently, 450 µL of chloroform: isoamyl alcohol (24:1) were added to each tube and vigorously mixed for 5 min. The tubes were centrifuged at 13,200 rpm for 5 min, the aqueous phase was transferred to a new tube, and 45 µL of 3 M sodium acetate and 1 mL of cold 100% ethanol were added to each sample, manually shaken, and kept at −20 °C minimum for 1 h. Tubes were centrifuged again at 13,200 rpm for 15 min. The supernatant was discarded and the pellet was air dried and resuspended in 100 µL of TE pH 7.5, incubated at 4 °C overnight and finally stored at −20 °C until use.

### 2.6. Polymerase Chain Reaction (PCR) and Sequencing

For molecular identification, the internal transcribed spacer region (ITS) of rDNA (ITS1, 5.8S, and ITS2) was amplified. The oligonucleotides used for polymerase chain reaction (PCR) were ITS5 (5′-GGAAGTAAAAGTCGTAACAAGG-3′) and ITS4 (5′-TCCTCCGCTTATTGATATGC-3′) [26]. Amplification conditions were an initial denaturation cycle of 4 min at 95 °C, followed by 35 cycles of 1 min at 95 °C, 1 min at 55 °C, 2 min at 72 °C, and a final extension cycle of 10 min at 72 °C. The 60S ribosomal protein and mitochondrial cox1 and cox2 loci were also obtained for selected isolates in the *P. capsici* species complex using primers as follows: 60SL10_for (5′-GCTAAGTGTTACCGTTTCCAG-3′) and 60SL10_rev (5′-ACTTCTTGGAGCCCAGCAC-3′), amplification conditions were an initial denaturation cycle of 2 min at 95 °C, followed by 34 cycles of 1 min at 95 °C, 1 min at 55 °C, 2 min at 72 °C, and a final extension cycle of 10 min at 72 °C [24]; for COI, COIF-1 (5′-TCAWCWMGATGGCTTTTTTCAAC-3′) and COIR-1 Fm85Mod (5′-RRHWACKTGACTDATRATACCAAA-3′), amplification conditions were as described by Robideau et al. [27]; for cox2, FM35 (5′-CAGAACCTTGGCAATTAGG-3′) [28] and FM78 (5′-ACAAATTTCACTACATTGTCC-3′), amplification conditions were an initial denaturation cycle of 2 min at 95 °C, followed by 30 cycles of 1 min at 94 °C, 1 min at 55 °C, 30 s at 72 °C, and a final extension cycle of 5 min at 72 °C [29]. Amplified fragments were analyzed by electrophoresis on 1.5% agarose gels and visualized on an UVP, TFML-26 (Upland, CA, USA) Amplicons were purified with the Wizard SV Gel and PCR Clean-Up System kit (Promega, Madison, WI, USA) and sent to Macrogen, South Korea, for the sequencing of both strands.

### 2.7. Molecular Identification

The ITS rDNA region of the isolates under study was used for molecular species determination. The oligonucleotides used for sequencing were the same used for PCR. The obtained sequences were assembled and trimmed using BioEdit version 7.0.5 [30], creating consensus sequences. The sequences were deposited in GenBank, and the accession numbers are listed in Table 1. These sequences were compared with *Phytophthora* ex-types in GenBank using the BLASTn (https://blast.ncbi.nlm.nih.gov/Blast.cgi) [31] and IDphy *Phytophthora* online resources [1,24]. The Maximum Likelihood tree, including relevant taxa in clades 1, 2, 7, and 8, was inferred with the IQ-TREE 2.2.2 [32] and Bayesian tree using MrBayes 3.2.6 [33].

### 2.8. Pathogenicity Tests

Five isolates were selected for pathogenicity tests, particularly those from *Phytophthora* hosts reported for the first time worldwide (*Cestrum nocturnum* and *Solanum ovigerum*). Since plants of *S. ovigerum* were not available, the isolate obtained from this host was inoculated on chili plants, and comparisons were made with *P. capsici* species complex isolates obtained from *C. annuum*. Additionally, the pathogenicity of *Phytophthora* isolates obtained from *C. nocturnum* was tested.

Isolates PV30 and PV35 (host: *C. annuum*), identified as representing an undescribed species within the *P. capsici* species complex, and PV44 (*S. ovigerum*) and PV46 (*C. annuum*), identified as *P. capsici*, were inoculated on six serrano chili plants var. Camino Real and 12 ornamental chili plants, placed in six packs, and randomly distributed. The inocula were produced as stated in the paragraph of morphological characterization for induction of sporulation. Sporangia were produced within one to two weeks. To induce zoospore release, the Petri plates were placed at 4 °C for 30 min, followed by 30 min at room temperature (25 °C). Zoospores were counted with a Neubauer chamber. Inoculation was carried out with 10,000 zoospores at the base of 35- to 40-day-old plants. The controls were inoculated with water. Plants were placed in trays with water to keep the soil saturated for 24 h. Plants were kept in the greenhouse under natural lighting until symptoms appeared (22 days). The isolate identified as belonging to the provisional taxon *P*. sp. *pseudocapsici* obtained from *C*. *nocturnum* (PV31) was inoculated onto plants of this host following the protocol described in the preceding paragraph.

#### Re-Isolation of the Pathogen

From inoculated plants displaying symptoms of necrosis and wilting, isolations were made on a selective NARPH culture medium. When characteristic *Phytophthora* mycelium growth was observed, it was transferred to cornmeal agar medium to induce reproductive structure formation. Based on reproductive structures, the identification of the isolates was confirmed.

## 3. Results

### 3.1. Ornamental Plants Infected by Phytophthora

Nineteen *Phytophthora* isolates were obtained from diseased plants collected in eight surveyed nurseries located in the municipalities of Michoacán: Morelia (5), Tarímbaro (1), and Uruapan (1), and one plant from Mexico City (CDMX). The samples were derived from 13 genera of ornamental plants (Table 1).

### 3.2. Species Identified Through Molecular Approach

**Figure 1:**
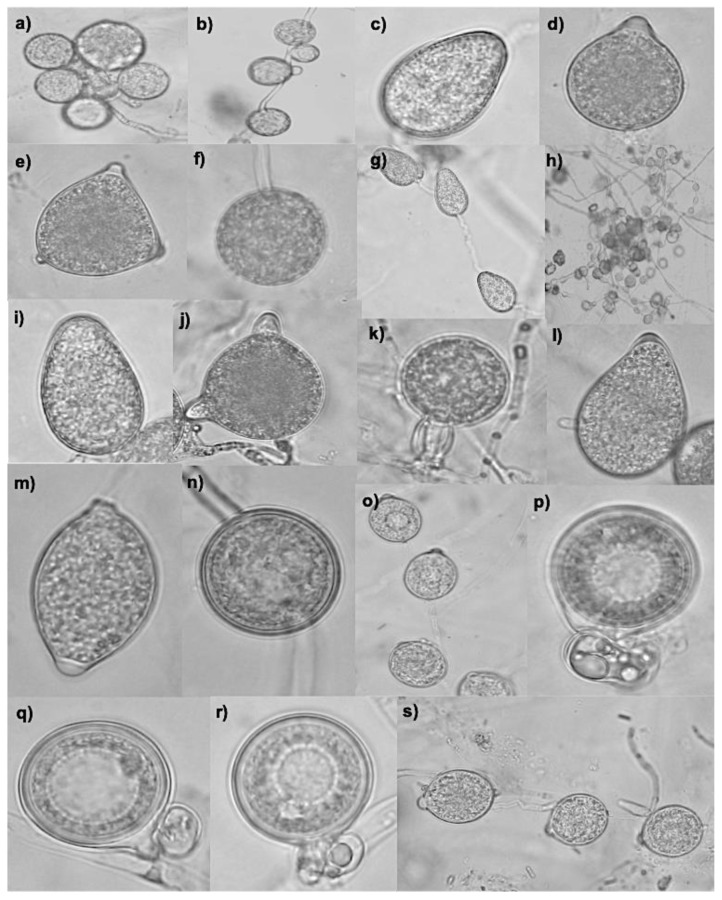
Morphological structures belonging to some *Phytophthora* species. (**a**–**c**) *P. cinnamomi*, (**a**) = globose chlamydospores; (**b**) = hyphal swellings; (**c**) = ovoid nonpapillated sporangia. (**d**–**f**) *P. nicotianae*, (**d**) = globose papillated sporangia; (**e**) = bipapillated sporangia; (**f**) = terminal globose chlamydospore. (**g**–**i**) *P. drechsleri*, (**g**) = simple sympodial sporangiophores; (**h**) = hyphal swellings; (**i**) = ovoid nonpapillated sporangia. (**j**–**l**) *P. capsici*, (**j**) = bipapillated sporangia; (**k**) = amphigynous antheridium; (**l**) = ovoid papillated sporangia. (**m**,**n**) *P. tropicalis*, (**m**) = limoniform sporangia; (**n**) = globose intercalary chlamydospore. (**o**–**s**) *P. cactorum*, (**o**,**s**) = simple sympodial sporangiophores; (**p**–**r**) = paragynous antheridium and aplerotic oospores. Observed under the light miscroscope at ×40 magnification.

**Figure 2:**
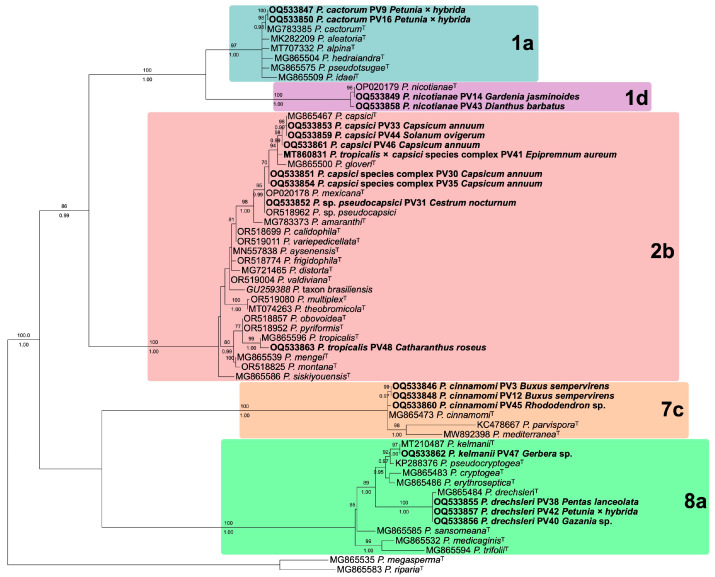
Maximum Likelihood ITS rDNA tree, inferred with IQ-TREE 2. Significant ultrafast bootstrap approximation values sit above branches, and posterior probabilities from a separate Bayesian analysis using MrBayes are below. Sequences from the current study are in bold and ex-type accessions are indicated (^T^).

**Figure 3:**
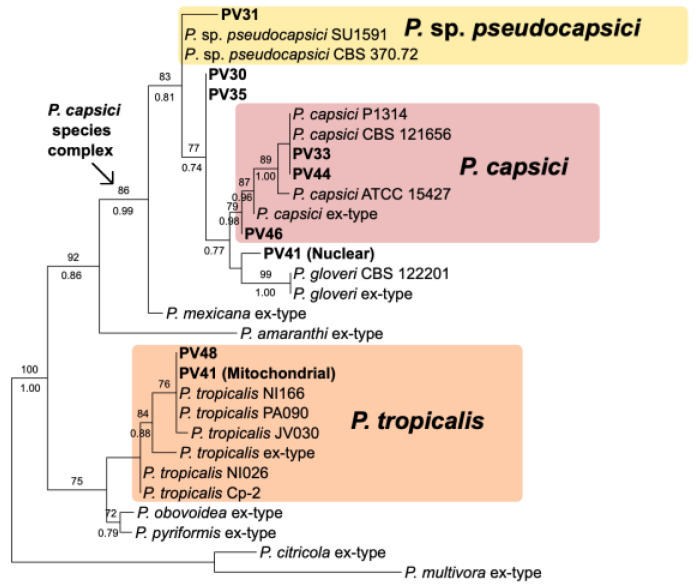
Maximum Likelihood tree from ITS rDNA, 60S ribosomal protein and mitochondrial cox2 loci, inferred with IQ-TREE 2. Significant ultrafast bootstrap approximation values sit above branches, and posterior probabilities from a separate Bayesian analysis using MrBayes are below. Sequence accessions are available in Supplementary Table S1.

Based on molecular identification through ITS sequences and morphological characteristics (Figure 1), the isolates obtained belong to the species *P. cactorum* (2), *P. capsici* (3), *P. cinnamomi* (3), *P. drechsleri* (3), *P. kelmanii* (1), *P. nicotianae* (2), *P. tropicalis* (1), and *P.* sp. *pseudocapsici* (1), (Table 1, Figure 2). Based on a three-locus phylogenetic analysis of the *P. capsici* species complex, two isolates (PV30 and PV35) appear to correspond to an undescribed member of the complex, while PV41 appears to represent an interspecific hybrid between another undescribed member of the complex and *P. tropicalis* (Figure 3). *Phytophthora* sp. *pseudocapsici* was identified in a new host, *C. nocturnum*. Both homothallic and heterothallic species were listed in Table 1, with a predominance of heterothallic species.

### 3.3. Pathogenicity Tests

#### Inoculation of *P. capsici* and *P.* sp. *pseudocapsici*

In ornamental chili plants, *P.* sp. *pseudocapsici* isolates caused 100% mortality in the inoculated plants; in the case of *P. capsici*, all the isolates caused 100% mortality, except for the one from *S. ovigerum* (PV44), which caused mortality in only 8% of the plants. In serrano chili plants, the isolates were virulent, causing 100% plant mortality, except for PV44, which showed no pathogenicity. *P.* sp. *pseudocapsici* induced wilting in the inoculated plants of *C. nocturnum*.

## 4. Discussion

Symptoms such as shoot rot, root and crown rot, dieback, or wilting were observed in the sampled hosts, caused by various *Phytophthora* species in ornamental plants from nurseries, as previously reported in other studies [34,35,36]. There are numerous reports worldwide about the damage caused by *Phytophthora* species to ornamental plants in nurseries [35,37,38]; however, in Mexico, studies on these *Phytophthora* phytosanitary problems remain scarce [12,13,14]. Therefore, the report on the *Phytophthora* species found in this study is relevant, since Mexico has a significant production of ornamental plants [10,21] and megadiverse vegetation [15]. It has been documented in several studies that *Phytophthora* has been spread from nurseries to other environments where it is not present, putting the natural ecosystems at high risk [2,16,39,40,41]. Regarding this issue, *Phytophthora* has already been found in urban gardens in Mexico [12], as well as, for the first time in the present investigation, in a home garden.

Although sampling was conducted in a limited number of nurseries, 19 isolates were identified from 13 genera of ornamental plants, indicating the problem that this pathogen represented in nurseries and the need for more extensive studies. Ten species were clustered in four clades (1, 2, 7 and 8): *P. cactorum*, *P. capsici*, *P. cinnamomi*, *P. drechsleri*, *P. kelmanii*, *P. nicotianae*, *P. tropicalis*, and two additional taxa in the *P. capsici* complex, with *P. kelmanii* being reported for the first time in Mexico [13,42]. Although this study was conducted during 2009 and 2010, most of the species are still prevalent in ornamental nurseries in Mexico [43]. The range of hosts is broad for some species found, such as *P. cactorum*, *P. capsici*, *P. cinnamomi*, *P. drechsleri*, and *P. nicotianae* [9,17,24,44], highlighting the importance of taking preventive measures when introducing infected plants from other states. This could prevent the spread of these oomycetes to areas where it was not previously present, affecting urban areas, natural ecosystems, and agricultural crops in Michoacán, an important state for its vegetation diversity and crop production [10,45]. The presence of *P. kelmanii*, identified for the first time in Mexico, emphasizes the potential risk due to its wide range of hosts [24]. A more exhaustive sampling of nurseries in major production areas may reveal the dispersion of this pathogen throughout the country.

Heterothallic species predominated over homothallic ones, indicating that most of the species were not surviving as oospores; rather, they survived in the form of mycelia or, in some cases, as chlamydospores. However, in one nursery, both compatibility types of *P. drechsleri* were found, suggesting that sexual reproduction might be occurring. Previous reports in Mexico mentioned the presence of both compatibility types of these species in the same plant or nursery soil [14]. One heterothallic species identified was *P. cinnamomi*, associated with wilting symptoms in *B. sempervirens* and *Rhododendron*. The detected compatibility type (A2) corresponded to what had been found in avocados in Mexico [46]. Despite no evidence of sexual reproduction in avocados, this species produces abundant chlamydospores, allowing it to survive for extended periods [47].

*Cestrum nocturnum* is reported for the first time as a host for *Phytophthora* worldwide. Detection of a member of the *P. capsici* species complex in ornamental chili (*Capsicum annuum*) is also a recently reported development. The homothallic nature of isolates from *C. nocturnum* is in accordance with Hotson and Harge’s (1923) report [48], while the heterothallic nature of isolates from ornamental chili matched other reports [1,49,50], indicating potential variation within the species complex. Abundant oospore production was observed in green bean–squash agar but not in other media [23]; therefore, this media is suggested as an option for oospore production.

Another novel host available globally is *S. ovigerum*. Further isolations are necessary due to its different behavior and phylogenetic distinction (Figure 3) compared to other *P. capsici* isolates. It did not cause symptoms in serrano chili, and, in ornamental chili, it exhibited lower virulence than other analyzed *P. capsici* isolates. Several plants have been reported for the first time as hosts for *Phytophthora* in Mexico, including *Buxus sempervirens* (boxwood), *Dianthus barbatus* (Sweet William), *Epipremnum aureum* (devil’s ivy or pothos), *Gardenia jasminoides* (gardenia), *Pentas lanceolata* (pentas), and *Rhododendron* (azalea). *Phytophthora drechsleri* infecting petunia in Mexico was previously confirmed [14]. Detection of more than one species within the same nursery or the same species infecting more than one host indicates potential cross-infections. Knowledge of the diversity of *Phytophthora* species affecting plants in Mexican nurseries with great heterogeneity shows the urgent need to improve strategies for managing diseases caused by these oomycetes.

Knowledge of the *Phytophthora* species affecting ornamental plants in Mexican nurseries is vital for plant producers, researchers, and regulators. This study contributes valuable information regarding the diversity of *Phytophthora* species in this productive system. Such insights may facilitate timely management of diseases caused by this oomycete and limit their spread to novel areas. Transport of plants from the nursery production areas to different Mexican states, such as Michoacán, which is the most important agricultural producer in Mexico, presents a growing threat.

## Figures and Tables

**Table 1:** *Phytophthora* isolates obtained from ornamental plants in nurseries located in different municipalities of Michoacán and one collected in Mexico City. Hosts and symptoms of the plants sampled identified through the ITS region sequencing, the subclades that they belong to, their respective compatibility types, and the accession number in GenBank (NCBI).

Isolate	Host	Symptoms	Locality	Species of *Phytophthora*	Subclade	Compatibility Type	Accession Number GenBank ITS
PV3	*Buxus sempervirens* L.	Wilt	Morelia, Michoacán (N1) *	*P. cinnamomi*	7c	A2	OQ533846
PV9	*Petunia* × *hybrida* Vilm.	Crown and root rot	Morelia, Michoacán (N1)	*P. cactorum*	1a	H	OQ533847
PV12	*Buxus sempervirens* L.	Wilt	Morelia, Michoacán (N2)	*P. cinnamomi*	7c	A2	OQ533848
PV14	*Gardenia jasminoides* J. Ellis	Wilt	Morelia, Michoacán (N2)	*P. nicotianae*	1d	A2	OQ533849
PV16	*Petunia* × *hybrida* Vilm.	Crown and root rot	Morelia, Michoacán (N3)	*P. cactorum*	1a	H	OQ533850
PV30	*Capsicum annuum* L.	Wilt	Tarímbaro, Michoacán (N5)	*P. capsici* species complex	2b	A1	OQ533851
PV31	*Cestrum nocturnum* L.	Wilt	Tarímbaro, Michoacán (N5)	*P.* sp. *pseudocapsici*	2b	H	OQ533852
PV33	*Capsicum annuum* L.	Wilt	CDMX (N7)	*P. capsici*	2b	A1	OQ533853
PV35	*Capsicum annuum* L.	Wilt	Tarímbaro, Michoacán (N5)	*P. capsici* species complex	2b	A1	OQ533854
PV38	*Pentas lanceolata* Forssk.	Dieback	Uruapan, Michoacán (N6)	*P. drechsleri*	8a	A2	OQ533855
PV40	*Gazania* sp. Gaertn.	Wilt	Uruapan, Michoacán (N6)	*P. drechsleri*	8a	A1	OQ533856
PV41	*Epipremnum aureum* (Linden and André) G. S. Bunting	Wilt	Tarímbaro, Michoacán (N5)	*P. tropicalis* × *capsici* species complex	2b	A2	MT860831
PV42	*Petunia* × *hybrida* Vilm.	Wilt	Uruapan, Michoacán (N6)	*P. drechsleri*	8a	A1	OQ533857
PV43	*Dianthus barbatus* L.	Crown and root rot	Uruapan, Michoacán (N6)	*P. nicotianae*	1d	A2	OQ533858
PV44	*Solanum ovigerum* Dunal.	Wilt	Morelia, Michoacán (N1)	*P. capsici*	2b	A1	OQ533859
PV45	*Rhododendron* sp. L.	Wilt	Morelia, Michoacán (N2)	*P. cinnamomi*	7c	A2	OQ533860
PV46	*Capsicum annuum* L.	Wilt	Morelia, Michoacán (N2)	*P. capsici*	2b	A1	OQ533861
PV47	*Gerbera* sp. L.	Wilt	Morelia (N8)	*P. kelmanii*	8a	A2	OQ533862
PV48	*Catharanthus roseus* (L) G. Don.	Shoot rot	Morelia, Michoacán (N4)	*P. tropicalis*	2b	ND	OQ533863

* = number of nursery, ND = Not determined

## Data Availability

The original contributions presented in the study are included in the article/Supplementary Materials; further inquiries can be directed to the corresponding author.

## Supplementary Materials

The following supporting information can be downloaded at: https://www.mdpi.com/article/10.3390/jof11030187/s1, Table S1: Additional loci sequences to support species determinations. within the *P. capsici* species complex for isolates PV-30, PV-35, PV-33, PV-44, PV-46, PV-48, PV-41 and PV-31.
